# Management of Panurethral Strictures by Preputial Graft Urethroplasty: Outcomes From a Tertiary Care Centre

**DOI:** 10.7759/cureus.82678

**Published:** 2025-04-21

**Authors:** Venkata Krishnan S, Deerush Kannan Sakthivel, Mukkani Velan, Nitesh Jain, Narasimhan Ragavan, Sandeep Bafna

**Affiliations:** 1 Urology, Apollo Hospitals, Chennai, IND; 2 Urology, Miami Cancer Institute Baptist Health South Florida, Miami, USA

**Keywords:** buccal mucosa graft, panurethral stricture, prepucial graft, urethral stricture, urethroplasty

## Abstract

Objective

The aim of the study was to review our outcomes with preputial graft urethroplasty for panurethral strictures in a small subset of the Indian population.

Patients and methods

We conducted a retrospective review of case records of the patients who had undergone preputial graft urethroplasty in our center from July 2022 to July 2023. Parameters such as uroflowmetry and maximal urine flow rate (Qmax) before surgery and at 1 month, 6 months, and 1 year after surgery, International Index of Erectile Function (IIEF) before and after surgery, intraoperative length of stricture, intraoperative time, length of hospital stay, postoperative course and complications and recurrences were recorded and analyzed. This spiral prepuceal graft was developed from preputial skin 5 cm wide, which is harvested in the shape of a helix and can reach up to 25 cm in length.

Results

A total of 15 patients were included in this study. All 15 patients were chronic smokers and pan chewers and hence were not ideal candidates for buccal mucosal graft. Four patients had previously undergone buccal mucosal urethroplasty. The preoperative mean Qmax was 4.99 ± 1.70 mL/s. At the time of catheter removal at 1 month, Qmax improved significantly to 19.37 ± 4.16 mL/s (t = 10.412, p <0.001). After 1 year, the mean postoperative Qmax decreased slightly to 17.37 ± 5.03 mL/s (t = 3.196, p = 0.006) but remained higher than the pre-surgery levels. Five patients had Clavien Dindo Grade I complications such as penile edema (n=1), scrotal hematoma (n=1), epididymorchitis (n=1), penile skin necrosis (n=1), and fever (n=1). None of the patients had recurrence at a follow-up of 1 year.

Conclusion

The preputial spiral graft urethroplasty is a safe procedure for panurethral strictures, those with previous urethral surgeries, those with unsuitable oral mucosa, and an alternative to buccal mucosal graft urethroplasty and two-stage urethroplasty.

## Introduction

Panurethral stricture disease is defined as the involvement of the entire length of the urethra, from the meatus to the bulbar urethra. Common etiologies include lichen sclerosus and iatrogenic causes such as traumatic catheterization and endoscopic instrumentation [1].

While strictures involving the anterior urethra are relatively common, panurethral strictures pose significant challenges to urologists. The use of buccal mucosa in urethroplasty, first described by Humby in 1941 [2], gained popularity for this indication in the early 1990s. It is now considered the gold standard urethral substitute due to several advantages, including ease of harvesting and handling, resistance to infection, a thick epithelium and thin lamina propria that facilitate early inosculation, compatibility with moist environments, and reduced donor site morbidity compared to lip or lingual mucosa [3].

The use of preputial skin is preferable to other graft materials such as lingual or buccal mucosa, as it avoids the need for a secondary oral procedure and associated complications at the donor site, such as bleeding, difficulty in oral intake, prolonged healing time, and contractures. Single-stage procedures utilizing penile skin are now favored over traditional buccal mucosal grafting (BMG), which is associated with significant donor site morbidity [4].

In this study, we evaluate the outcomes of spiral preputial graft urethroplasty, as described by Kulkarni et al. [5], in a cohort of 15 patients with panurethral stricture disease.

## Materials and methods

**Figure 1:**
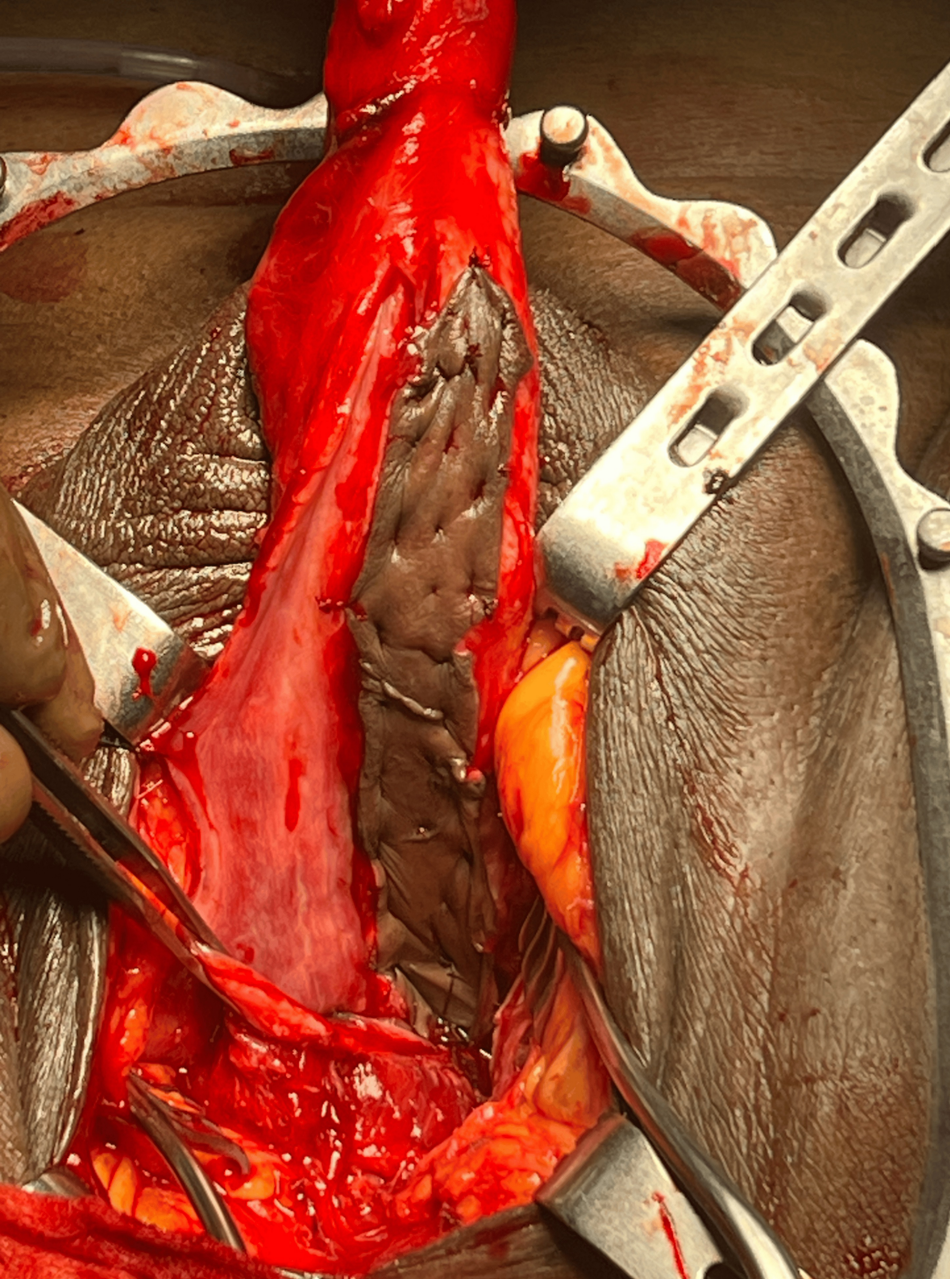
Prepucial graft quilted over the urethra

**Figure 2:**
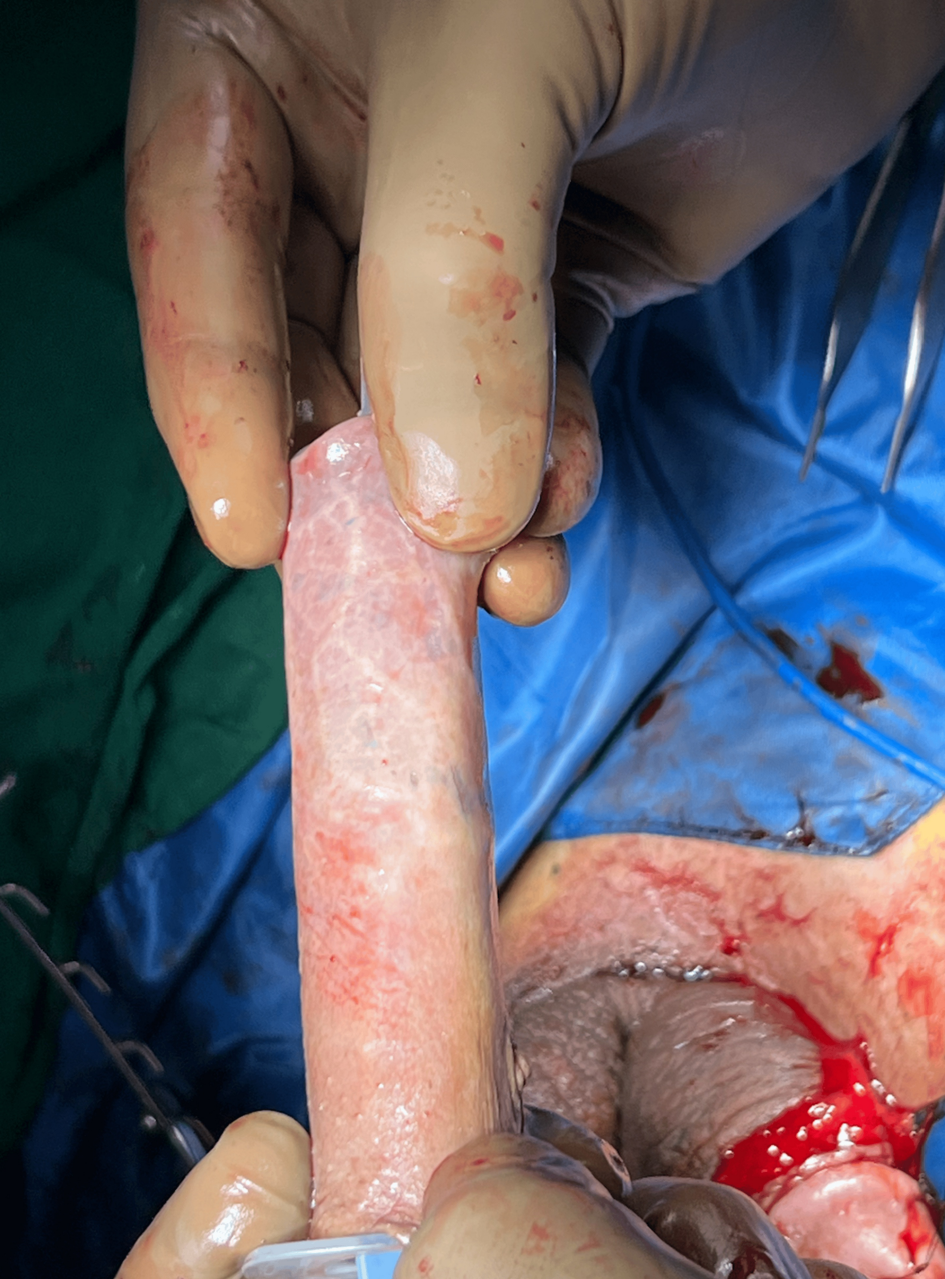
Prepucial graft harvested and rolled over a plastic 10 cc syringe

The study adhered to the guidelines outlined in the Declaration of Helsinki and received approval from the Institutional Ethics Committee - Bio Medical Research, Apollo Hospitals, Chennai (application number AMH-C-S-095/J 0-24). Case records of 15 patients who underwent preputial graft urethroplasty were selected for analysis. Written informed consent was obtained from all patients as part of the preoperative process. All procedures were performed by a single surgeon with extensive expertise in reconstructive urology, having performed over 250 urethroplasties annually. Data collected included patient age, etiology of stricture, history of previous urethroplasty, dorsal visual internal urethrotomy (DVIU), and dilatation. Preoperative assessments included uroflowmetry and maximal urine flow rate (Qmax), as well as the International Index of Erectile Function (IIEF). The length and nature of strictures were determined by ascending urethrograms. Intraoperative parameters such as stricture length and operative time were also recorded. Postoperative data collected included hospital stay duration, duration of antibiotic therapy, duration of Foley catheter placement, date of catheter removal, uroflowmetry, and Qmax at the time of catheter removal, at 6 months, and at 1 year. IIEF scores post surgery, complications, and recurrence rates were also documented. The surgical technique followed was based on the previously described Kulkarni technique (Figure 1 and Figure 2).

Postoperative care and follow-up

Patients received intravenous antibiotics for 2 days postoperatively, followed by oral antibiotics for 28 days. Follow-up included scheduled clinical visits and uroflowmetry assessments. The first follow-up occurred on postoperative day 5 for perineal wound evaluation. The next follow-up, for catheter removal, was scheduled 30 days after surgery and included a uroflowmetry assessment. Subsequent follow-up visits were scheduled at 3 months, 6 months, and 1 year, during which both clinical evaluation and uroflowmetry were performed.

Definition of treatment success

Treatment success was defined as the absence of patient-reported symptoms along with a urinary flow rate exceeding 12 mL/sec. Patients with persistent symptoms, a Qmax below 12 mL/sec, or recurrent urinary tract infections were further evaluated with ascending urethrograms to confirm stricture recurrence.

## Results

A total of 15 patients were included in this study (Table 1). Four patients had previously undergone buccal mucosal urethroplasty before presenting to our center. The majority (n=7) of patients were aged 60 years and older, comprising 46.7%. The mean age was 59 years, with an interquartile range (IQR) of 53-70 years.

**Table 1:** Case-wise description of etiology, preoperative Qmax, postoperative Qmax, and recurrence UTI - urinary tract infection, BMG - buccal mucosal graft, NA - Not available

Case no	Stricture etiology	Pre-operative Qmax	Post-operative Qmax at 1 year	Complications	Recurrence	Treatment for recurrence
1	Recurrent UTI	NA	10	Nil	Yes	Dilatation
2	Lichen	4.8	18	Penile skin necrosis	No	-
3	Instrumentation	6.6	16	Nil	No	-
4	Failed BMG	7	17.2	Scrotal hematoma	No	-
5	Idiopathic	6	22	Nil	No	-
6	Idiopathic	5.5	11	Nil	Yes	Dilatation
7	Idiopathic	5.4	14	Nil	No	-
8	Recurrent UTI	6.2	15	Nil	No	-
9	Recurrent UTI	7.3	17.3	Nil	No	-
10	Idiopathic	4.3	19.5	Fever	No	-
11	Idiopathic	3.2	24.9	Epididymorchitis	No	-
12	Lichen	1.8	15	Nil	No	-
13	Idiopathic	2.7	28	Penile edema	No	-
14	Instrumentation	NA	12	Nil	No	-
15	Failed BMG	4.1	20.6	Nil	No	-

The most common etiology (n=6) of stricture was idiopathic, accounting for 40% of cases. Four patients (26.67%) had a history of prior urethroplasty, and in this subset, the use of a buccal graft was not considered. All 15 patients were chronic smokers and pan chewers, further limiting the suitability of buccal mucosal grafts.

Most surgeries (n=8) lasted between 150 and 200 minutes, with a mean operative time of 185 minutes (IQR: 170-195 minutes). Postoperative hospital stay was typically limited to just 2 days in most (n=14) of the patients.

Preoperative Qmax was 4.99 ± 1.70 mL/s. At the time of catheter removal, Qmax significantly improved to 19.37 ± 4.16 mL/s. At the last follow-up, Qmax slightly declined to 17.37 ± 5.03 mL/s but remained significantly higher than the preoperative values. The mean stricture length was 10.42 ± 1.68 cm (IQR: 9-12 cm). The mean International Index of Erectile Function (IIEF) score preoperatively was 21.21 ± 3.45, which dropped to 16.71 ± 3.15 postoperatively. Minor complications (Clavien-Dindo Grade I) were observed in five patients (33.33%), including epididymo-orchitis, fever, penile edema, penile skin necrosis, and scrotal hematoma, each affecting one patient. No stricture recurrence was noted at the 1-year follow-up.

## Discussion

Management of panurethral strictures has been a conundrum for urologists over the decades. Challenges include deciding between single-stage versus staged procedures, availability of sufficient tissue for reconstruction, and the potential for associated complications [6].

Several single-stage techniques have been described to address these complex strictures [4,5,7]. The ingenious Q-flap technique described by Morey et al. utilizes a distal circumferential penile skin flap for reconstruction of panurethral strictures and is known for its long, vascularized pedicle. However, it is limited by prolonged operative time and complications such as scrotal hematoma, recurrent strictures, and, in rare cases, cerebrovascular accidents [8].

Preputial skin offers several advantages: it is thin, pliable, hairless, located close to the urethra, and provides adequate graft material for long-segment strictures. Moreover, it withstands prolonged exposure to urine better than other skin substitutes [4].

Harvesting the preputial skin in a spiral shape was first described by Kulkarni et al. and allows the creation of longer grafts. This mucocutaneous graft, derived from a 5-cm-wide preputial skin strip, can be extended up to 20 cm when harvested in a helicoidal fashion. The technique improves anterior urethral exposure, reduces the risk of diverticulum formation, and avoids the oral complications associated with buccal mucosal grafts (BMG) [5].

Kulkarni also described a one-stage dorsal onlay BMG urethroplasty technique via a perineal incision. While this technique offers a high success rate and a reduced risk of fistula formation and hypospadiac meatus, it carries the risk of recurrence at the dual-graft anastomotic site, narrowing at the suture line, the requirement for healthy oral mucosa, and the burden of oral complications [7].

Two patients in our study with previously failed BMG urethroplasty achieved good functional outcomes following spiral graft urethroplasty.

The results of spiral preputial graft urethroplasty in our cohort were consistent with the outcomes reported by Kulkarni et al. [5]. Although the operative time was slightly longer compared to other single-stage penile skin techniques [4,9], the complication rate was lower, and the technique proved successful even in complex cases, including those with previous urethroplasty failure.

With newer and innovative robotic approaches for reconstruction gaining popularity in urology and reconstructive surgery, the exploration of alternative techniques such as the spiral preputial graft paves the way for future advancements in the field [10,11,12]. 

The limitations of our study include a small sample size and a relatively short follow-up duration. This limits the ability to assess late stricture recurrence, long-term graft contraction, and the effects on micturition and erectile function.

## Conclusions

Preputial spiral graft urethroplasty is a safe and effective option for managing complex panurethral strictures, particularly in patients with prior failed surgeries or unsuitable oral mucosa. It offers adequate graft length, favorable tissue characteristics, and minimal donor-site morbidity. Our early results are encouraging, with good functional outcomes and low complication rates. Larger studies with long-term follow-up are needed to validate its wider applicability.
